# Chemically linked phage idiotype vaccination in the murine B cell lymphoma 1 model

**DOI:** 10.1186/1479-5876-11-267

**Published:** 2013-10-23

**Authors:** Tim Roehnisch, Cornelia Then, Wolfgang Nagel, Christina Blumenthal, Todd Braciak, Mariel Donzeau, Thomas Böhm, Carole Bourquin, Fuat Oduncu

**Affiliations:** 1 Division of Hematology and Oncology, Medizinische Klinik und Poliklinik IV, Klinikum der Universität München, Ziemssenstrasse 1, D-80336, Munich, Germany; 2 Division of Endocrinology and Diabetology, Medizinische Klinik und Poliklinik IV, Klinikum der Universität München, Munich, Germany; 3 Helmholtz Zentrum München, Deutsches Forschungszentrum für Gesundheit und Umwelt, Munich, Germany; 4 Maître de conférences (MCF), Université de Strasbourg, Unité UMR Biotechnologie et Signalisation Cellulaire, Strasbourg, France; 5 Département de Médecine, Chair of Pharmacology, Université de Fribourg, Fribourg, Switzerland

**Keywords:** Phage idiotype vaccination, B cell lymphoma, Murine BCL1 lymphoma model, KLH

## Abstract

**Background:**

B cell malignancies are characterized by clonal expansion of B cells expressing tumor-specific idiotypes on their surface. These idiotypes are ideal target antigens for an individualized immunotherapy. However, previous idiotype vaccines mostly lacked efficiency due to a low immunogenicity of the idiotype. The objective of the present study was the determination of the feasibility, safety and immunogenicity of a novel chemically linked phage idiotype vaccine.

**Methods:**

In the murine B cell lymphoma 1 model, tumor idiotypes were chemically linked to phage particles used as immunological carriers. For comparison, the idiotype was genetically expressed on the major phage coat protein g8 or linked to keyhole limpet hemocynanin. After intradermal immunizations with idiotype vaccines, tolerability and humoral immune responses were assessed.

**Results:**

Feasibility and tolerability of the chemically linked phage idiotype vaccine was demonstrated. Vaccination with B cell lymphoma 1 idiotype expressing phage resulted in a significant survival benefit in the murine B cell lymphoma 1 protection model (60.2 ± 23.8 days vs. 41.8 ± 1.6 days and 39.8 ± 3.8 days after vaccination with wild type phage or phosphate buffered saline, respectively). Superior immunogenicity of the chemically linked phage idiotype vaccine compared to the genetically engineered phage idiotype and keyhole limpet hemocynanin-coupled idiotype vaccine was demonstrated by significantly higher B cell lymphoma 1 idiotype-specific IgG levels after vaccination with chemically linked phage idiotype.

**Conclusion:**

We present a novel, simple, time- and cost-efficient phage idiotype vaccination strategy, which represents a safe and feasible therapy and may produce a superior immune response compared to previously employed idiotype vaccination strategies.

## Background

Anti-tumor vaccines hold out the prospect of effective tumor therapies with minimal side effects. A successful example is the anti-CD20 antibody rituximab acting as passive vaccination against B cell lymphoma. However, rituximab targets CD20 in general, thus depleting not only B cell lymphoma cells but also normal B cells [1]. It is envisioned that a personalized active vaccination strategy targeting tumor-specific antigens may evoke an even better and more sustained therapeutic response. An ideal and easily identifiable tumor-specific antigen is the variable region of the clonal immunoglobulin (idiotype, Id) expressed on the surface of B cell malignancies, being unique to each neoplastic B cell clone.

The effectiveness of Id vaccines largely depends on a sufficient immunogenicity of the Id, which represents a tumor-specific antigen [2], but nevertheless is a self-protein. For the purpose of provoking immunogenicity, the Id is usually coupled to a strong immunogenic carrier protein, such as keyhole limpet hemocyanin (KLH), and co-administered with immunostimulatory adjuvants, mainly granulocyte-monocyte colony stimulating factor (GM-CSF) [3,4]. Despite these procedures, Id-based immunotherapy has so far resulted in mostly disappointing clinical outcomes and clinical phase III studies aimed at obtaining regulatory approval for Id-KLH vaccines failed to reach their primary endpoints [5,6]. With the aim of enhancing the idiotype immunogenicity, we utilized the immunogenic properties of the filamentous phage, which is more typically employed in phage display technology as a powerful molecular tool for antibody engineering [7]. Peptides displayed on the surface of filamentous phage are able to induce humoral as well as cell-mediated immune responses [8], making phage particles an attractive antigen delivery system [9]. We here present a novel chemically linked phage Id vaccine characterized by a higher Id density on the phage surface compared to previously used genetically engineered phage vaccines.

## Methods

### Purification of BCL1-IgM

The hybridoma cell line 123 F6 was used as source for mouse anti-BCL1 IgM (LGC Standards). Cells were kept in complete Dulbecco’s Modified Eagle Medium with 10% (v/v) fetal calf serum, 10^4^ IU/ml Penicillin and 10 mg/ml Streptomycin (Gibco) at 37°C and 5% CO_2_. Mouse BCL1-IgM was purified from the supernatant employing protein A chromatography followed by ion exchange chromatography on an ÄKTA Purifier 10 using Unicorn 4.11 software (Amersham Biosciences) with modifications in accordance with Reichart et al. [10]. Samples (500 μl) were passed through 0.8 μm and 0.2 μm nitrocellulose filters and equilibrated with 500 μl 20 mM tetra-sodium diphosphate buffer (pH 6.4; Merck, Darmstadt, Germany) at room temperature for 10 minutes and then bound to a HiTrap Protein A HP/5 ml column (Amersham Biosciences) equilibrated with binding buffer (100 mM sodium citrate/150 mM NaCl/pH 6.4; Merck). After removal of impurities with binding buffer, IgM fractions were eluted using a pH step gradient (100 mM sodium citrate/150 mM NaCl/pH 3.5). Samples were collected in tubes containing 100 μl 1 M Tris/HCl/pH 9.5. The collected IgM pool was dialyzed against 20 mM Tris/HCl/pH 8.5 and bound to HiTrap Q-HP/5 ml column (Amersham Biosciences) equilibrated with 20 mM Tris/HCl/pH 8.5. Samples were eluted with 20 mM Tris/1 M NaCl/pH 8.5 using a linear salt gradient and the paraprotein was dialyzed against phosphate-buffered saline (PBS; Invitrogen, Karlsruhe, Germany). The purified protein was sterile filtered through a 0.2 μm nitrocellulose; protein concentration was determined by spectrophotometry at 280 nm.

### Preparation of Id vaccines

The preparation of bacteriophages (M13K07, Amersham Biosciences) at large scale was performed according to standard methods [11]. For harvesting bacteriophages, the cell suspension was subjected to centrifugation (15 minutes, 4°C, 7,000 rpm). The supernatant was transferred to 1/5 volume of 5 × PEG/NaCl solution (20% (w/v) PEG 6,000/2.5 M NaCl in water) and incubated at 4°C for 2 h to precipitate bacteriophages. After centrifugation (15 minutes, 4°C, 7,000 rpm), the resulting pellet was suspended in PBS and centrifuged again (15 minutes, 4°C, 10,000 rpm). The resulting supernatant was repeatedly precipitated with PEG/NaCl solution.

Contaminating endotoxins were removed by repeated two-phase Triton X-114 separation as described previously [12], resulting in a reduction of endotoxins to a concentration of < 1 endotoxin unit/ml as determined by the Limulus Amebocyte Lysate QCL-1000 Assay. The purified bacteriophages were passed through a 0.4 μm nitrocellulose filter.

Recombinant Id-phage carrying the murine BCL1 Id protein was produced by phagemid rescue employing M13 helper phage (M13K07, Amersham Biosciences). The single-chain variable fragment-BCL1 [13] was fused to the g8 protein of the M13 phage particle bearing a His tag at the carboxy-terminus. Subsequently, a phage designated BCL1-g8 was generated and purified employing the technologies of Apalexo Biotechnology as described [14].

Chemically linked Id-phage designated BCL1-WT was generated by coupling purified BCL1 to bacteriophages. 500 μl of a 0.1% (v/v) glutaraldehyde/water solution was added drop wise to 1.5 ml bacteriophage solution (50 mg/ml in PBS) during slow vortexing until an Id protein/phage ratio of 1:10 (w/w) was achieved. The resulting mixture was incubated for 1 hour at 25°C with agitation at 1600 rpm. The reaction was stopped by adding 100 μl 1 M glycine/PBS (w/v) for 1 hour at 25°C with agitation at 1600 rpm. Samples were dialyzed against PBS and passed through a 0.4 μm nitro cellulose filter. Chemically linked BCL1-KLH was generated accordingly, resulting in a conjugate with an Id/KLH ratio of 1:10 (w/w).

### Mouse vaccinations

Animal experiments were conducted in accordance with the European Union Laws and Guidelines and were approved by the local ethics committee (Ethikkomission der Medizinischen Klinik, Munich University) and governmental authorities (J.-Nr. 211-2531-22/98). Nine week old BALB/c mice were purchased from Elevage Janvier and kept under standardized pathogen-free conditions. For preclinical investigation of phage Id-vaccines, the murine BCL1 lymphoma model was employed [15].

Firstly, BCL1 tumor cells (LGC Standards) were cultivated by injection of 10^6^ BCL1 cells into BALB/c mice intraperitoneally. After four weeks, approximately 10^8^ BCL1 tumor cells were harvested from the spleen and were characterized by flow cytometry and ELISA. Isolated cells were found to express IgM and lambda light chain, whereas surface expression of IgG, IgG1, IgG2a or kappa light chains was not detected (data not shown).

Secondly, mice were subcutaneously injected with 10^10^, 10^11^ or 5 × 10^11^ bacteriophages or 0.25 mg BCL1-KLH with or without addition of 20 μg granulocyte-macrophage colony stimulating factor (GM-CFS; Leukomax®, Novartis) as indicated. Vaccinations were carried out weekly for four weeks.

Thirdly, for examination of vaccine efficacy, 10^5^ BCL1 tumor cells were administered intraperitoneally at day 7 following the last immunization. After challenge with tumor cells, the spleen usually becomes infiltrated with lymphoma cells leading to splenomegaly and mice typically develop leukemia and die within 35 days of injection [13,15,16]. Mice were monitored by triweekly physical examination including the palpatory determination of the spleen size. Termination criteria were a spleen index > 3 according to Vitetta [17] and signs of distress.

### Enzyme-linked immunosorbent assay (ELISA)

Assay plates with 96 wells (Costar®, ImmunoChemistry Technologies) were coated with the respective capture antibodies (100 μl/well) at 4°C over night, blocked with PBS plus 1% (v/v) bovine serum albumin (Sigma Aldrich) and washed three times with PBS, 0.1% (v/v) Tween 20 (Sigma Aldrich). Sera were added to the wells in PBS dilutions of 1/200 or 1/1000 as indicated. Subsequently, the plate was incubated for 1 hour at 37°C and washed three times with PBS plus 0.1% (v/v) Tween 20 before 100 μl of a 1/1000 PBS-dilution of HRP-conjugated detection antibodies (goat-anti-mouse IgG/IgG1/IgG2a, goat-anti-rat IgG or rat-anti-mouse IgM/lambda/kappa, Southern Biotech) were added to each well for two hours at 37°C. After washing three times with PBS, 0.1% (v/v) Tween 20, 100 μl of 2,2′-Azinobis [3-ethylbenzothiazoline-6-sulfonic acid]-diammonium salt substrate (Thermo Fisher Scientific) was added for 20 minutes before stopping the reaction with 100 μl of 1% (w/v) sodium dodecyl sulfate in PBS (Merck). Sample concentrations were determined by spectrophotometry at 405 nm.

### Flow cytometry

Flow cytometry analyses were conducted with a Coulter Epics XL-MCL flow cytometer (Beckman). Control-Fluorescein isothiocyanate (−FITC), CD4-FITC, Control-phycoerythrin (−PE), CD8-PE, CD45-FITC, CD3-PE, CD4-PE, CD19-PE, kappa-FTC, lambda-FITC and IgG-, IgG1-, IgG2a-, IgM-FITC were purchased from Beckmann Coulter and Southern Biotech.

### Statistics

Data are shown as means +/− standard deviation. Significance was declared for p-values < 0.05 as assessed by Student’s T-test and log-rank test for survival of mice.

## Results

### Determination of the optimal phage dosage

**Figure 1:**
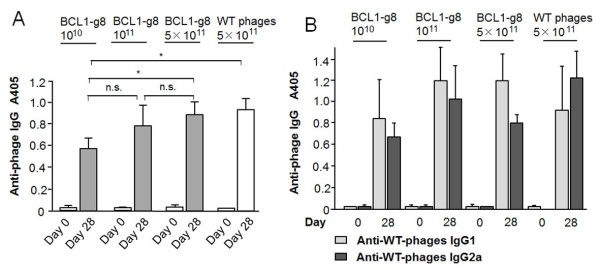
**Determination of the phage vaccine dosage.** The humoral immune response after vaccination of mice with phage was determined by ELISA (antibody dilution: 1/1000). **(A)** Total IgG response at day 28 was measured after the last vaccination with 10^10^, 10^11^ or 5 × 10^11^ phage particles weekly for 4 weeks (n = 6). **(B)** Anti-phage specific IgG isotype levels before and at day 28 after 4 weekly vaccinations with 10^10^, 10^11^ or 5 × 10^11^ phage particles (n = 6). * p > 0.05.

To evaluate the effect of phage dosage with regard to humoral anti-phage responses that might provide guidance for determining effective vaccination dosing, mice were vaccinated weekly with 10^10^, 10^11^ or 5 × 10^11^ phage particles for 4 weeks and anti-phage IgG responses were evaluated at day 28 after the last vaccination. Phage-specific antibodies were induced in a dose-dependent manner with significantly higher IgG levels after application of 5 × 10^11^ compared to 10^10^ phages (Figure 1A). No differences were found between 5 × 10^11^ BCL1-g8 and wild-type phage. Considering each IgG isotype IgG1 and IgG2a individually revealed no significant increase in the isotypes after a higher dose of the BCL1-g8 vaccine (Figure 1B).

### Tumor protection conferred by Id-phage vaccines

**Figure 2:**
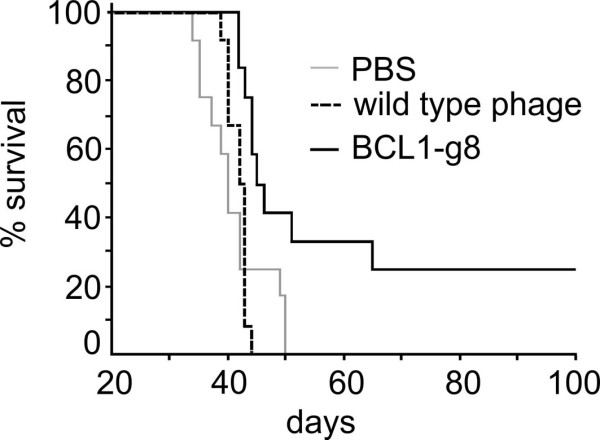
**Tumor protection procured by Id-phage vaccines.** Kaplan-Meier plot of the survival of the mice vaccinated weekly with 5 × 10^11^ bacteriophages (BCL1-g8 or PBS or wild type phage as control). Seven days after the last vaccination, 10^5^ BCL1 cells were injected intraperitoneally; n = 12. p < 0.05 (BCL1-g8 vs. wild type phage and vs. PBS).

In order to explore the feasibility of employing bacteriophages in a vaccination strategy in the murine BCL1 lymphoma model, we designed recombinant BCL1-phage vaccines for use as a potential therapeutic agent. BALB/c mice (n = 12/group) were vaccinated weekly with 5 × 10^11^ BCL1-expressing or wild-type bacteriophages (BCL1-g8, wild-type phage) or PBS. Seven days after the last vaccination, 10^5^ BCL1 lymphoma cells were injected intraperitoneally. Survival of mice vaccinated with BCL1-expressing phage was significantly longer (60.2 ± 23.8 days) compared to mice vaccinated with wild-type bacteriophages serving as a control (41.8 ± 1.6 days) or PBS (39.8 ± 3.8 days) (Figure 2; p < 0.05). All animals in the control treatment groups were deceased by day 45, while 50% of the BCL1-g8 vaccinated animals were still surviving. A complete tumor protection (survival without any clinical sign of tumor growth until the end of the 100 day observation period) was observed in 25% of the BCL-g8 vaccinated mice.

### Humoral immune response after vaccination of mice

#### Anti-phage humoral immune response

**Figure 3:**
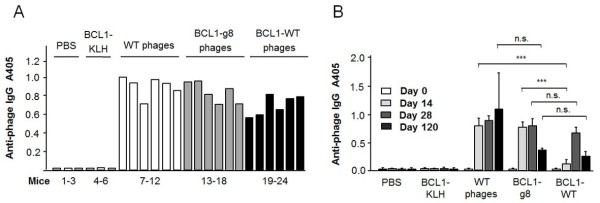
**Anti-phage IgG response.** The humoral immune response after vaccination of mice with phage was determined by ELISA (antibody dilution: 1/1000). **(A)** Total IgG response of single mice 4 weeks (day 28) after the last of 4 weekly vaccinations with PBS, BCL1-KLH, BCL1-g8, BCL1-WT or wild type phage (n = 6). **(B)** Sustainability of the humoral immune response to phage vaccines: Anti-phage IgG levels before (day 0) and at day 14, 28 and 120 after the last of 4 weekly vaccinations with BCL1-g8, BCL1-WT or wild type phage (5 × 10^11^ each; n = 6). * p > 0.05, *** p < 0.001.

We next examined the immunogenic properties of the phage vaccine with respect to humoral immunity. The induction of phage-specific IgG was compared for wild-type phages, recombinant BCL1-expressing phages (BCL1-g8) and wild-type phages chemically coupled to the BCL1 protein (BCL1-WT). As shown in Figure 3A, mice vaccinated with either wild-type phage or BCL1-g8 and BCL1-WT revealed similar anti-phage IgG levels with small inter-individual variations and a trend towards lower IgG levels after vaccination with BCL1-WT compared to wild type phage.

To determine the sustainability of the anti-phage humoral response, total IgG levels were determined at day 14, 28 and 4 months (day 120) after the last of 4 weekly vaccinations. Figure 3B shows the time course of the total anti-phage IgG response. All phage vaccinations induced anti-phage IgG responses at each of the measuring time points. Notably, the phage-specific IgG response was significantly weaker at day 14 in the BCL1-WT group as compared to the BCL1-g8 group. However, at day 28 and 120, anti-phage IgG levels were similar for both groups. With regard to the induction of anti-phage IgG isotype levels, a similar pattern with lower IgG1 and IgG2a levels was observed in the BCL1-WT group at day 14 compared to the BCL1-g8 group, but again, similar levels were detected in both groups by day 120 (data not shown). Anti-phage IgG levels were lower for both the BCL1-g8 and BCL1-WT vaccines at day 120 in comparison to wild-type phage.

**Figure 4:**
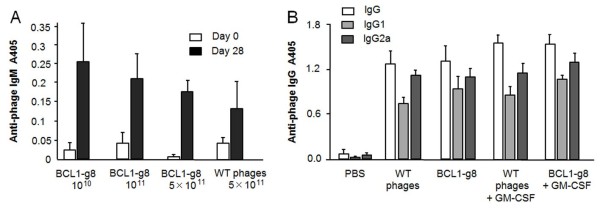
**Anti-phage IgM response and IgG response with or without addition of GM-CSF. (A)** IgM responses at day 28 were measured after the last vaccination with 10^10^, 10^11^ or 5 × 10^11^ phage particles weekly for 4 weeks (n = 6; antibody dilution: 1/200). **(B)** Anti-phage specific IgG isotype levels at day 28 after 4 weekly vaccinations with 5 × 10^11^ phage particles with or without addition of 20 μg GM-CSF (n = 6; antibody dilution: 1/1000).

There was a non-significant trend towards lower anti-phage IgM levels with increasing vaccination dose (Figure 4A). Addition of 20 μg GM-CSF (s.c.) to vaccinations with 5 × 10^11^ BCL1-g8 or wild type phage did not induce significantly higher total anti-phage IgG, IgG1 or IgG2a plasma levels (Figure 4B).

#### Anti-Id humoral immune response

**Figure 5:**
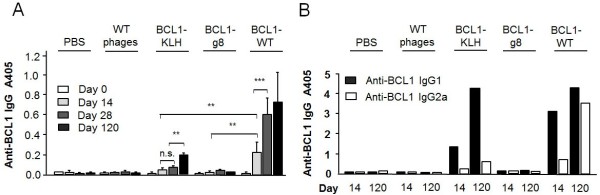
**Anti-phage IgG response.** The anti-phage humoral immune response after vaccination of mice with phage was determined by ELISA (antibody dilution: 1/1000) **(A)** Anti-BCL1-specific IgG response before (day 0) and at day 14, 28 and 120 after vaccination with PBS, wild type phage, BCL1-KLH, BCL1-g8 or BCL1-WT (5 × 10^11^ each; n = 6). **(B)** Anti-BCL1-specific IgG isotypes at day 14 and 120 after vaccination. * p > 0.05, ** p < 0.01; *** p < 0.001.

Most importantly for the determination of any potential benefit in our vaccine’s effects, we next measured anti-Id-specific humoral responses generated in response to the phage vaccines. Figure 5A shows the anti-BCL1-specific IgG responses at day 14, 28 and 120 after vaccination with 5 × 10^11^ BCL1-g8 or BCL1-WT phage and in comparison to BCL1-KLH treatment. Strikingly, BCL1 Id-specific IgG levels were found to be significantly higher in the BCL1-WT group as compared to the BCL1-KLH and the BCL1-g8 group at all time points, indicating the superiority of the BCL1-WT vaccine formulation. Moreover, preliminary data indicates higher IgG2a anti-Id levels after BCL1-WT compared to BCL1-KLH vaccination at day 14 and 120 (Figure 5B).

## Discussion

The chemically linked phage Id vaccine represents a fast and cost efficient method for generating tumor-specific vaccinations at high yield quantities. In the murine BCL1 lymphoma model, feasibility and tolerability of this phage Id vaccination strategy was demonstrated. Phage particles offer an excellent antigen delivery system and a powerful tool for vaccine design [18], as peptides expressed as recombinant fusions with either the minor coat protein g3 or the major coat protein g8 [19,20] can be easily presented to the immune system as part of the coat protein. In order to utilize the powerful phage-inherent immunogenicity for Id vaccination, we took advantage of the phage’s feature to allocate thousands of well-defined sites available for chemical conjugation [21] and chemically coupled the high molecular weight BCL1 Id to the phage surface at a high density.

Investigation of the resulting phage vaccine composition revealed a superior immunogenicity of the novel phage vaccine formulation in comparison to previously used Id vaccination regimens. Id-specific antibody levels were significantly higher and rose earlier after vaccination with the novel phage vaccine as compared to vaccination with the BCL1-KLH and the BCL1-g8 vaccine. Thus, although KLH-coupled Id proteins still represent the gold standard for Id vaccinations and have been shown to provide protection in the murine tumor challenge model [22,23], chemically conjugated Id-phage seems to have the potential of a superior immune reaction. The observation that the novel phage Id composition obtained by chemical coupling induced lower phage-specific IgG responses as compared to the BCL1-g8 hybrid phage formulation is possibly due to both a higher Id density and chemical crosslinking of the BCL1-WT phages, thus covering phage-specific antigens against B cell recognition.

Besides the induction of Id-specific antibodies, cellular immune response is also of importance for producing an effective anti-tumor response. In treatment-naive patients with indolent B cell lymphoma, only cellular-mediated responses correlated with superior progression-free survival and durable objective remissions [24]. In multiple myeloma patients, the cellular immune response is especially crucial, since myeloma cells secrete their tumor-specific immunoglobulins and thus the anti-Id humoral immune response may result in binding and neutralizing of anti-Id specific antibodies by soluble paraproteins [25]. Accordingly, reduction of circulating myeloma cells correlates with vaccine-induced Id-specific T cell responses [26]. In this context, it is fundamental that phages are able to be incorporated by antigen-presenting cells and to induce not only a humoral, but also a cellular immune response [8,27]. Uptake and processing of the phage-coupled Id protein and subsequent presentation via both the MHC I and II is very likely. Antibody subtype analysis in the phage-vaccinated mice revealed that Id-KLH induced mainly the IgG1 isoform. In contrast, BCL1-WT vaccination induced a more predominant IgG2a response, which is considered to be favorable for lymphoma protection [22,28]. IgG2a is associated with Th1 responses in mice, which are related to better T cell-mediated immune response and tumor killing [29]. Furthermore, antibody-mediated cellular cytotoxicity is mainly dependent on IgG2a [28,30] and IgG2a was shown to yield a better anti-lymphoma effect [22].

Most Id vaccination studies use GM-CSF as adjuvant to induce a stronger cellular immune response, presumably by recruitment of dendritic cells [3,4]. However, the use of GM-CSF in Id-vaccination is controversial, as on the one hand GM-CSF might be important for inducing a T cell response to Id vaccination, which is supported by the fact that GM-CSF in combination with IL-12 leads to a stronger induction of an Id-specific T cell response than IL-12 alone in myeloma patients [31,32]. On the other hand, GM-CSF was shown to induce growth-stimulating effects on human myeloma cells *in vitro*[33] and extramedullary progression of multiple myeloma [34]. In the present study, GM-GSF did not induce a stronger antibody response or a survival benefit compared to phage Id vaccination alone, suggesting that the strong immunogenicity of the phage vaccine formulation itself is sufficient to induce an effective immune response without the use of an additional adjuvant. Thus, omission of GM-CSF adjuvant may be worth considering in future studies employing phage vaccine formulations.

Further strategies to improve the Id immunogenicity are under investigation. With regard to the role of phage as a carrier for future vaccine formulations, phage may be further optimized by design, for example by co-expression of fragments or antigenic determinants, which promote the uptake of bacteriophages by dendritic cells and thus the tumor-protective capacity [35]. On the other hand, genetic removal or modification of immunodominant regions of coat proteins was demonstrated to focus and improve the epitope-specific immune response by decreasing the antigen complexity of the phage surface [36].

A strength of the current study is the comprehensive assessment of the humoral immune response against a novel phage-Id formulation in comparison to the previously known genetically engineered Id-phage and the gold standard Id-KLH using the established ELISA method. The major limitation is that we did not directly examine the cellular immune response. Furthermore, we employed the murine BCL1 lymphoma model as a model for B cell malignancies with paraprotein secretion and clinical trials in myeloma patients are needed to confirm the tolerability and efficacy of the novel vaccine in humans.

## Conclusions

While refinements for increasing the immunogenicity of the target Id may still be needed, we conclude from our data that Id proteins chemically conjugated to phage particles appear to be suitable for use as vaccines. The immune stimulatory potential of the chemically linked phage Id-vaccine appears to be superior to the gold standard Id-KLH vaccine and to genetically engineered phage vaccines. Based on the encouraging immune responses generated in the murine BCL1 lymphoma model, we believe that clinical trials including patients with B cell malignancies are warranted to confirm the therapeutic efficiency of this approach.

## Abbreviations

BCL: B cell lymphoma; ELISA: Enzyme-linked immunosorbent assay; g3: Minor coat protein g3; g8: Major coat protein g8; GM-CSF: Granulocyte-monocyte colony stimulating factor; HRP: Horseradish peroxidase; Id: Idiotype; Ig: Immunoglobulin; IL: Interleukin; KLH: Keyhole limpet hemocynanin; M: Mol/liter; MHC: Major histocompatibility complex; NaCl: Sodium chloride; n.s.: Not significant; PBS: Phosphate-buffered saline; rpm: Round per minute; PEG: Polyethylene glycol; s.c.: Subcutaneously; SDS-PAGE: Sodium dodecyl sulfate polyacrylamide gel electrophoresis.

## Competing interests

The authors declare that they have no competing interests.

## Authors’ contributions

TR conceived of the study, planned its design and coordination and analyzed the data. CT analyzed the data and wrote the manuscript. WN participated in the design of the study, analyzed the data and helped to draft the manuscript. CB performed parts of the experiments. TB analyzed the data and helped to draft the manuscript. MD and TB participated in the data collection and analysis. CB performed parts of the experiments and analyzed the data. FO planned the study design, managed and coordinated the process of data analysis and drafting of the manuscript. All authors read and approved the final manuscript.
